# Inter-organ metabolic interaction networks in non-alcoholic fatty liver disease

**DOI:** 10.3389/fendo.2024.1494560

**Published:** 2025-01-09

**Authors:** Yu-Hong Fan, Siyao Zhang, Ye Wang, Hongni Wang, Hongliang Li, Lan Bai

**Affiliations:** ^1^ State Key Laboratory of New Targets Discovery and Drug Development for Major Diseases, Gannan Innovation and Translational Medicine Research Institute, Gannan Medical University, Ganzhou, China; ^2^ State Key Laboratory of New Targets Discovery and Drug Development for Major Diseases, Gannan Innovation and Translational Medicine Research Institute, Ganzhou, China; ^3^ Department of Cardiology, Renmin Hospital, Wuhan University, Wuhan, China; ^4^ Medical Science Research Center, Zhongnan Hospital of Wuhan University, Wuhan, China; ^5^ State Key Laboratory of New Targets Discovery and Drug Development for Major Diseases, Gannan Innovation and Translational Medicine Research Institute, Key Laboratory of Prevention and Treatment of Cardiovascular and Cerebrovascular Diseases, Ministry of Education, Gannan Medical University, Ganzhou, China

**Keywords:** non-alcoholic fatty liver disease, Inter-organ crosstalk, fatty acid synthesis, mitochondrial homeostasis, endoplasmic reticulum (ER) stress

## Abstract

Non-alcoholic fatty liver disease (NAFLD) is a multisystem metabolic disorder, marked by abnormal lipid accumulation and intricate inter-organ interactions, which contribute to systemic metabolic imbalances. NAFLD may progress through several stages, including simple steatosis (NAFL), non-alcoholic steatohepatitis (NASH), cirrhosis, and potentially liver cancer. This disease is closely associated with metabolic disorders driven by overnutrition, with key pathological processes including lipid dysregulation, impaired lipid autophagy, mitochondrial dysfunction, endoplasmic reticulum (ER) stress, and local inflammation. While hepatic lipid metabolism in NAFLD is well-documented, further research into inter-organ communication mechanisms is crucial for a deeper understanding of NAFLD progression. This review delves into intrahepatic networks and tissue-specific signaling mediators involved in NAFLD pathogenesis, emphasizing their impact on distal organs.

## 1 Introduction

Non-alcoholic fatty liver disease (NAFLD) is a metabolic disorder characterized by excessive fat accumulation in the liver in the absence of alcohol or other known liver toxins. The disease progresses through various stages: from simple hepatic steatosis (NAFL) to nonalcoholic steatohepatitis (NASH), then to liver fibrosis, cirrhosis, and potentially hepatocellular carcinoma (HCC) (1). Current definitions of NAFLD emphasize the exclusion of other causes of liver disease and the presence of specific liver histopathological changes. Growing understanding of NAFLD pathophysiology highlights its strong link to metabolic dysregulation, with obesity, type 2 diabetes mellitus (T2DM), and hyperlipidemia as key risk factors. Recognizing these metabolic underpinnings, a 2022 consensus favored the term “metabolic dysfunction-associated fatty liver disease” (MAFLD) (2)to more comprehensively reflect the disease’s etiology. However, due to varying study aims and scope, the literature continues to utilize both NAFLD and MAFLD designations (3). This review, therefore, does not prioritize a specific terminological definition but rather examines the broader range of evidence related to NAFLD/MAFLD.

The increasing prevalence of NAFLD is linked to lifestyle factors, including physical inactivity and high caloric intake. As treatments for hepatitis C improve and cases of hepatitis B decrease, NAFLD is anticipated to become a leading cause of liver-related health issues and mortality, with predictions indicating it may surpass other conditions as the primary reason for liver transplantation by 2030 (4). Currently, therapeutic options are limited to dietary changes and exercise, with preclinical pharmacological interventions showing only modest efficacy or notable side effects. Although the FDA approved Resmetirom for NAFLD treatment in late 2023, its Phase III clinical data demonstrated only up to 30% effectiveness (5, 6). Therefore, there is an immediate necessity to broaden and thoroughly comprehend the mechanisms underlying NAFLD, as well as to identify practical therapeutic targets or interventions for its treatment.

The primary driver of fat accumulation in NAFLD is excessive dietary intake of sugars and fats. This abnormal fat buildup impairs lipid metabolism and transport, leading to the production of lipotoxic substances that trigger stress responses in hepatocyte organelles. These metabolic disturbances also affect other organs, altering their metabolism and prompting them to release signaling factors that influence NAFLD progression. NAFLD is recognized as a multisystem metabolic disorder (7), associated with various comorbidities including obesity, T2DM, hypertension, dyslipidemia, atherosclerotic cardiovascular disease (ASCVD), and chronic kidney disease (CKD) (8–11).

This review delves into the intrahepatic network mechanisms underlying NAFLD pathogenesis and examines the roles of tissue-specific signaling mediators, emphasizing their impact on distant organs throughout NAFLD development and its complications. The discussion will begin with adipose tissue, closely linked to NAFLD pathophysiology, and extend to other critical tissues such as the intestine, skeletal muscle, and endocrine pancreas.

## 2 Methods

The purpose of this literature review was to identify comprehensive evidence that elucidates a network of inter-organ metabolic interactions in NAFLD, with a particular emphasis on the most recent data published within the last 3 years. To achieve this, we conducted a search on PubMed on September 1, 2024, utilizing the following filters:

Key Words: (NAFLD or MAFLD) AND (Adipose); (NAFLD) AND (Brain); (NAFLD) AND (Gastrointestinal tract); (NAFLD) AND (Pancreas); (NAFLD) AND (Bone); (NAFLD) AND (Skeletal muscle); (NAFLD) AND (Exercise).

Article type: Clinical Trial, Meta-Analysis, Randomized Controlled Trial, Review, Systematic Review.

Species: human, animals.

Age: child, adult male, adult female, elderly.

Publication date: within 3 years; Accepted PubMed articles outside of 3 years if they demonstrated strong relevance to the review’s objectives.

Inclusion criteria: Disease burden and epidemiology of NAFLD, Intrahepatic network mechanisms associated with the development of NAFLD, Association of NAFLD with other metabolic disorders and factors influencing its progression, Impact of NAFLD development on extrahepatic organs, Role of novel biomarkers indicating crosstalk between extrahepatic organs in NAFLD, Interventions targeting systemic metabolic dysfunction related to NAFLD.

Quality and limitations of cited studies.

Quality: Rigorous selection criteria were employed to ensure the inclusion of high-quality studies; Attention was paid to the most recent research data from the last 3 years, capturing the latest advancements in the field; The wide range of studies covered, including clinical trials, meta-analyses, and systematic reviews, provided a wealth of information for a comprehensive understanding of the relationships between organs in metabolic diseases associated with NAFLD.

Limitations: Many studies relied on preclinical models (e.g., mouse and rat models) to explore the correlation between NAFLD and metabolic factors, and the results of these models may not be fully applicable to humans, with certain limitations; due to the diverse types of included studies, there may be heterogeneity in study design, sample size, and research methodology, which may result in controversial findings; The inclusion of articles published only in English may have excluded high-quality studies in other languages, which may lead to a certain impact on the comprehensiveness of the findings; although only the latest research progress in the past 3 years has been included, due to the large number of NAFLD-related studies and the limited space, the authors may not be able to be as in-depth and detailed in discussing the individual studies, which may lead to certain important points or findings not being fully elaborated or verified.

### 2.1 Intrahepatic network and metabolic signaling in NAFLD

**Figure 1:**
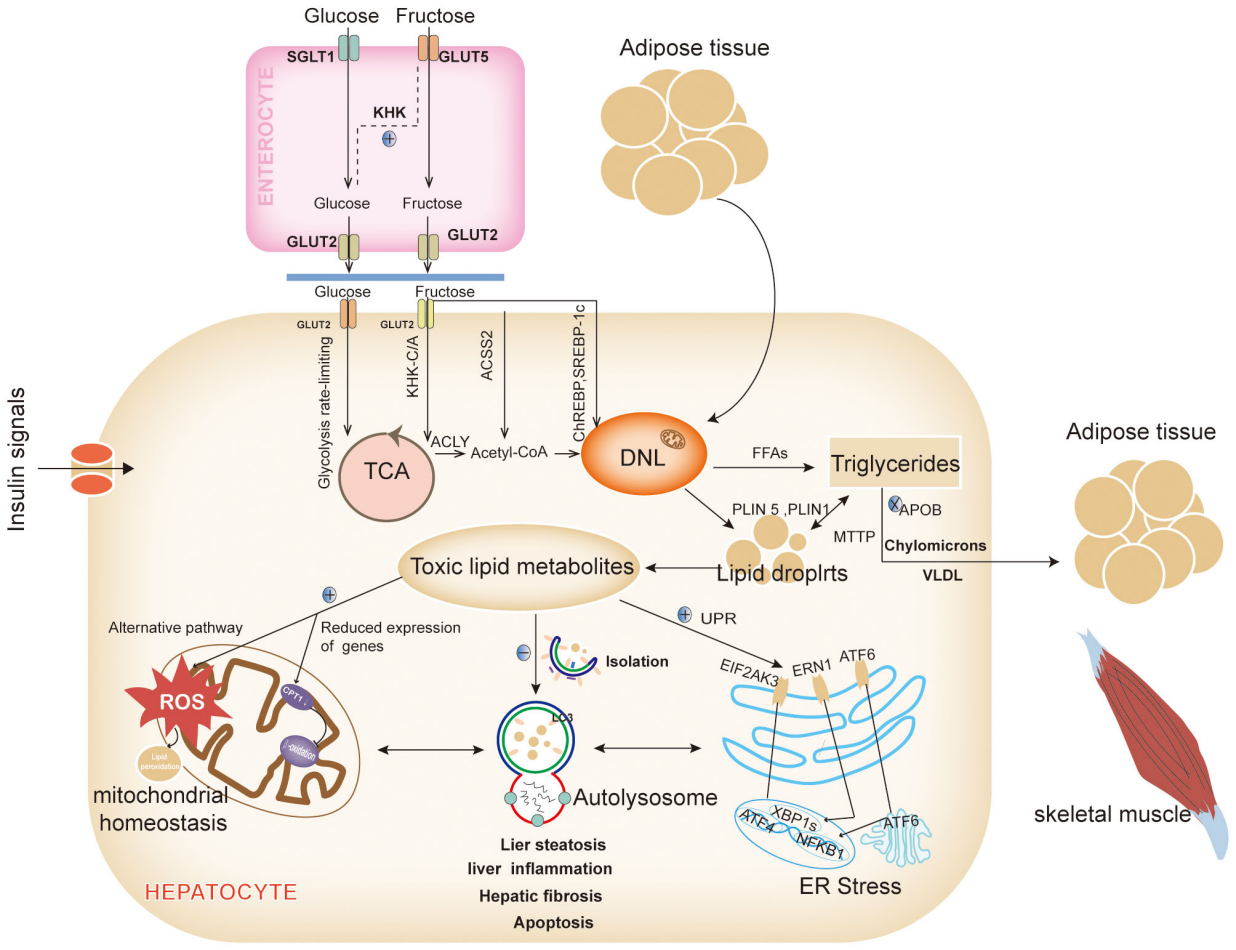
Intrahepatic network and metabolic signaling in NAFLD. The most important reason for the alteration of the metabolic signaling network in the liver is the abnormalities in the hepatic lipid synthesis pathway and/or lipid metabolism pathway, which leads to the abnormal accumulation of intrahepatic lipids, the generation of large amounts of lipotoxic substances in the liver, affects multiple metabolic pathways, disrupts the mitochondrial homeostasis, inhibits the clearance of lipid autophagy, and promotes the ER stress. DNL, Lipid *de novo* synthesis; GLUT 2, Glucose transporter 2; TCA, The tricarboxylic acid; KHK-C/A, Ketohexose kinase - C/A; ACLY, ATP citrate lyase; Acetyl-CoA, Acetyl-coenzyme a; CPT1α, Carnitine palmitoyltransferase 1a; ACSS2, Acetyl-CoA synthetase 2; ChREBP, Carbohydrate-responsive element-binding proteins; LDs, lipid droplets; VLDLs, Very low-density lipoproteins; MTTP, Microsomal TAG transport protein; APOB, Apolipoprotein B; FFAs, Free fatty acids; PLIN, Perilipin; LC3, Light chain 3; UPR, Unfolded protein response; EIF2AK3(PERK), Eukaryotic translation initiation factor 2 alpha kinase 3; ATF 6, Activated transcription factor 6; ERN1(IRE1), ER to nucleus signaling 1; NFKB, Nuclear factor kappa B; XBP1s, X-box binding proteins 1; ROS, Reactive oxygen species (Drawing by Adobe Illustrator 2020).

The excessive accumulation of lipids in the liver is a primary driver of NAFLD. Lipids, which act as pivotal signaling molecules across various pathways, are implicated in this process. Aberrant fat deposition in the liver not only contributes to hepatic inflammation and fibrosis but also disrupts the function of intrahepatic organelles and has detrimental effects on extrahepatic organs (12). Therefore, a thorough understanding of the origins and ultimate destinations of fatty acids within the liver is essential (**Figure 1**).

#### 2.1.1 Fatty acid synthesis

Under normal dietary and metabolic conditions, intrahepatic fatty acids primarily originate from dietary lipids and the mobilization of adipose tissue. However, in states of overnutrition or metabolic imbalance, dietary carbohydrates significantly contribute to the development of NAFLD. Although free saturated fatty acids (SFAs) more markedly increase intrahepatic triglyceride content compared to free sugars at equivalent intake levels (13), SFAs exert their effects mainly through adipose tissue lipids. In contrast, free sugars enhance hepatic lipid *de novo* synthesis (DNL). High-SFA foods are often perceived as greasy, making it challenging to consume them in quantities higher than free sugars (13).

Among free sugars, fructose, and glucose are the primary source materials for DNL. Glucose enters the bloodstream via the intestinal sodium-dependent glucose cotransporter protein 1 (14) and is subsequently transported to hepatocytes by the insulin-independent glucose transporter-2 (GLUT -2), where it is either stored as glycogen or metabolized for energy (14). Fructose, on the other hand, is particularly harmful. While glucose is regulated and undergoes a rate-limiting glycolytic process in hepatocytes (14), fructose can bypass these regulatory mechanisms (14). Low doses of fructose are metabolized to glucose in the intestines via GLUT-5 and then enter the liver as glucose (13). However, high doses of fructose exceed intestinal clearance capacity and are absorbed as fructose through the fructose-specific GLUT2. Inside the liver, fructose is phosphorylated by ketohexose kinase-C/A (KHK-C/A), leading to citric acid production through adenosine triphosphate (ATP) citrate lyase, which provides cytoplasmic acetyl-CoA for DNL (15, 16). Additionally, KHK-C upregulates acetylation of global proteins, including carnitine palmitoyltransferase 1α (CPT1α), thereby promoting lipogenesis and inhibiting lipolysis (17). High fructose intake also accelerates the provision of cytoplasmic acetyl-CoA for DNL via acetyl-CoA synthetase 2 (ACSS2) (16). Moreover, excess fructose metabolism depletes ATP, activates the adenosine monophosphate (AMP) deaminase pathway, and leads to overproduction of uric acid, which reduces fatty acid β-oxidation and further promotes DNL (18). Excess fructose activates various signaling pathways, such as carbohydrate-responsive element-binding protein (ChREBP), which in turn upregulates the expression of acetyl-CoA carboxylase (ACC), fatty acid synthase (FAS), and other enzymes involved in DNL (19).

Fructose also affects NAFLD development through extrahepatic organs. It increases intestinal epithelial permeability, allowing toxic microbial metabolites to enter the liver and activate inflammatory signals in hepatocytes and immune cells. Prolonged fructose intake exacerbates intestinal dysbiosis, which further amplifies inflammatory responses. Additionally, fructose affects the central nervous system, influencing neuronal and hormonal signals related to appetite and promoting increased food intake.

#### 2.1.2 Fatty acid metabolism

Fatty acids enter the liver through two primary pathways: 1) regulated by metabolic signals and oxidative processes, and 2) stored as lipid droplets (LDs). A small proportion of fatty acids entering the liver is utilized for energy through β-oxidation. However, the majority of these fatty acids respond to metabolic signals and are exported from the liver as triglycerides (TAGs) via celiac microparticles and very low-density lipoproteins (VLDLs). These lipoproteins facilitate the delivery of energy to peripheral tissues, including adipose tissue, skeletal muscle, and the heart. An abnormal accumulation of free fatty acids (FFAs) within the liver occurs when metabolic signals, such as those from insulin, glucagon, and thyroid hormones, are disrupted. Additionally, genetic defects, such as mutations in the microsomal triglyceride transport protein and apolipoprotein B, can inhibit the loading of TAGs into VLDLs, thereby impeding their export from the liver through apolipoproteins (20).

Excess fatty acids that are not oxidized for energy are stored in the liver as LDs after packaging by the ER. The liver’s capacity to store LDs is limited, and excessive accumulation leads to LDs lipolysis, which releases FFAs and results in inflammatory damage and hepatocyte death. Alterations in the structure and function of LDs are crucial in the development of NAFLD (21). For instance, overexpression of Perilipin (PLIN) 5, a Perilipin family protein associated with LD development, has been linked to increased fibrosis and apoptosis (22), while PLIN1 shifted-code variants have been associated with hepatic vascularization (23). Conversely, the function of LDs is closely related to mitochondria and autophagy, which are essential for maintaining lipid metabolism homeostasis (24).

#### 2.1.3 Lipid autophagy

Excess lipids are eliminated from the liver through the process of lipid autophagy, which prevents the abnormal accumulation of lipids. In response to *in vivo* signaling, LDs recruit light chain 3 (LC3), initiating the formation of restriction membranes via autophagy-related protein 7 (ATG7)-dependent mechanisms. These restriction membranes, known as autophagosomes, encapsulate the LDs and transport them to lysosomes for degradation (25). However, a high-fat diet (HFD) induces LC3 positivity in hepatic LDs, leading to a reduction in autophagic isolation (25). Chronic overnutrition diminishes hepatic ATG7 levels, thereby impairing autophagic activity (26). This impairment is evident in ATG7 knockout mice, which exhibit reduced lipolysis, up-regulated expression of thermogenic genes, and restored adipose tissue development in brown and inguinal adipose tissue (27). Autophagy in adipose tissue is critical for recruiting various cell types involved in fat storage, including neurons in the hypothalamus, foam cells derived from macrophages, lymphocytes, adipocytes, and gastrointestinal epithelial cells (24). Disruption of hepatic autophagy contributes to insulin resistance (IR) and exacerbates ER stress (26).

#### 2.1.4 ER stress

The ER is particularly susceptible to lipotoxicity, which can disrupt the regulation of the unfolded protein response (UPR). The UPR is orchestrated by three key transmembrane proteins located in the ER: eukaryotic translation initiation factor 2 alpha kinase 3 (EIF2AK3, also known as PERK), activating transcription factor (ATF) 6, and ER to nucleus signaling 1 (ERN1, also known as IRE1). These proteins collectively modulate cellular inflammation and apoptosis (28). EIF2AK3 facilitates the nuclear translocation of ATF4, thereby promoting autophagy. Concurrently, EIF2AK3 activates the phosphatidylinositide-3-kinase(PI3K) -AKT serine/threonine kinase 1 (AKT1) signaling cascade and cooperates with the ERN1-triggered 5’ AMP-activated protein kinase (AMPK) pathway to drive autophagy and inhibit apoptosis (29). ERN1 further mediates the translocation of mitogen-activated protein kinase 8 (MAPK8, also known as JNK1) and nuclear factor kappa B (NFKB), enhancing the expression of genes associated with apoptosis and inflammation. Additionally, ERN1 activates X-box binding protein 1, which triggers the c-Jun N-terminal kinase(JNK) and NFKB kinase subunit β (IKBKB)- NFKB1 signaling pathways, influencing inflammatory processes and reactive oxygen species production (29). ATF6, upon translocating to the Golgi, undergoes cleavage and subsequently translocates to the nucleus, where it upregulates genes involved in apoptosis, autophagy, and inflammation (28). Furthermore, ER stress induces angiopoietin-like protein 8, inhibiting lipoprotein lipase activity and promoting autophagy (30).

#### 2.1.5 Mitochondrial homeostasis

Mitochondria are the principal sites for fatty acid β-oxidation, with FFAs entering the mitochondria for oxidative catabolism via the CPT1 protein located in the outer mitochondrial membrane. Inhibition of CPT1 impairs fatty acid β-oxidation, leading to reduced degradation of FFAs. In the hypothalamus, AMPK phosphorylates ACC and lowers malonyl-CoA levels to regulate CPT1 activity. This regulatory axis has been explored as a target for therapeutic interventions in NASH (31). When FFAs overload occurs in the liver, the mitochondrial respiratory chain activity is upregulated, enhancing various specific fatty acid oxidation processes including propionic acid oxidation, unsaturated fatty acid oxidation, alpha-oxidation, and omega-oxidation. This upregulation leads to an overproduction of reactive oxygen species (ROS) beyond the capacity of normal antioxidant defenses, resulting in oxidative stress and progression towards NASH. Concurrently, the surplus ROS triggers lipid peroxidation, producing harmful byproducts such as 4-Hydroxynonenal and malondialdehyde, these compounds exacerbate mitochondrial dysfunction and damage plasma and intracellular membranes, contributing to cell necrosis (32). Researches have shown reduced expression of peroxisome proliferator-activated receptor γ (PPARγ)- coactivator 1α(PGC-1α), nuclear respiratory factor 1, and mitochondrial transcription factor A in the livers of individuals with NAFLD, indicating that mitochondrial number and function are crucial in NAFLD pathogenesis (33). Additionally, lysophosphatidylcholine acyltransferase 3 (LPCAT3) is significantly inhibited in human NASH livers. LPCAT3 deficiency increases the saturation of phospholipids in the mitochondrial inner membrane and heightens stress-induced mitochondrial autophagy, leading to decreased mitochondrial content, increased fragmentation, and subsequent inflammatory and fibrotic consequences associated with NASH (34).

### 2.2 Extrahepatic organ-specific network

#### 2.2.1 Adipose tissue

Changes in adipose tissue biology are pivotal in the onset of NAFLD, affecting both energy balance and immune modulation. Adipose tissue, as a key organ in inter-organ communication, releases a variety of signaling molecules that relay energy-related information to other tissues (35).

**Leptin:** Leptin, encoded by the Lepob gene and secreted by subcutaneous adipose tissue, regulates numerous aspects of energy metabolism. Elevated leptin levels are inversely related to NAFLD development (36). Leptin modulates NAFLD through several mechanisms: 1) It inhibits hypothalamic orexigenic neurons (Neuropeptide Y/Agouti-related protein, NPY/AgRP) and activates opioid Pro-opiomelanocortin (POMC)-expressing neurons, reducing appetite and energy intake (37). 2) It stimulates sympathetic signaling in white adipose tissue (WAT) and brown adipose tissue (BAT), enhancing thermogenesis and energy expenditure. Leptin also influences subcutaneous WAT lipolysis via the hypothalamic-pituitary-adrenal axis (38). 3) Leptin promotes glucose uptake in extrahepatic tissue (BAT, brain, heart, etc.) and reduces intrahepatic gluconeogenesis. A 2022 study demonstrated that leptin stimulates the export of hepatic VLDL-loaded triacylglycerols to adipose tissue via brain-vagus nerve-hepatic communication (39). 4) It regulates energy metabolism by binding to the leptin receptor, activating various signaling pathways including Janus Kinase 2 (JAK2)-Signal transducer and activator of transcription 3(STAT3), PI3K-AKT, and extracellular protein kinases pathways, with JAK2/STAT3 being particularly significant (40). Targeted deletion of JAK2 in liver cells prevents steatosis and glucose intolerance induced by HFD (41), while the absence of STAT3 results in increased feeding behavior and obesity (42). Elevated leptin levels are often associated with obesity, a known risk factor for NAFLD (43).

**Adiponectin:** This adipokine, secreted as lipocalin, has been shown to enhance insulin sensitivity, reduce inflammation, and in some cases, promote weight loss (44). Lipocalin signals primarily through lipocalin receptors 1 and 2 (AdipoR1 and AdipoR2). AdipoR1, predominantly expressed in skeletal muscle, interacts closely with the AMPK pathway. Its functional mechanisms include: 1) Activation of insulin receptor substrate 1/2, thereby engaging the PI3K/AKT pathway to enhance insulin sensitivity (44). 2) Activation of the p38 MAPK pathway, leading to GLUT-4 translocation in muscle cells and affecting muscle glucose utilization. 3) Hydrolysis of ceramide to sphingosine via the liver kinase B1 -AMPK pathway, which increases Sphingosine 1-phosphate (S1P) levels, restores impaired insulin signaling, and boosts lipid metabolism (45). 4) Reduction of oxidative stress and JNK signaling pathways via the AMPK-forkhead box protein O (FoxO) signaling axis, thereby counteracting hepatic steatosis (46). 5) Promotion of nitric oxide production via AMPK-activated endothelial nitric oxide synthase (eNOS), which induces vasorelaxation, and inhibition of IkappaB kinase/NFKB/Phosphatase and tensin homolog-induced apoptosis via AMPK activation (47). AdipoR2, mainly located in the liver, is associated with the PPARα pathway, enhancing fatty acid oxidation and energy expenditure through increased expression of acetyl coenzyme A oxidase and uncoupling proteins (48). Additionally, AdipoR2, like AdipoR1, exhibits ceramidase activity, facilitating ceramides hydrolysis. S1P, generated by sphingosine kinases 1 and 2, is activated through S1P receptor 3-sterol regulatory element-binding protein-1(SREBP1) and PPARγ pathways, upregulating stearoyl coenzyme A desaturase (SCD) and enhancing lipid metabolism (49). Unlike leptin, adiponectin levels rise with moderate weight gain but decrease with excessive obesity (50).

**Resistin:** Resistin, produced by WAT in mice and also observed in human preadipocytes and mature adipose tissues (51), is associated with IR and contributes to NAFLD, atherosclerosis, CVD, and CKD, as supported by an expanding array of research (52). Elevated resistin levels in NASH patients correlate with liver inflammation and fibrosis (53), likely due to its pro-inflammatory properties mediated through NFκB and MAPK pathways. Resistin activates the PI3K-AKT-NFKB cascade or directly stimulates NFKB, leading to the release of pro-inflammatory cytokines such as TNF-α, IL-1β, IL-6, and IL-12 (54). Additionally, stimulation of p38 MAPK and JNK enhances pro-inflammatory cytokines production, with p38 MAPK activation reducing eNOS levels and obstructing vasodilation (55). Recent findings indicate that variations in the resistin gene may influence the risk of developing NAFLD (56).

**Other adipokines**: Adipsin (complement factor D) is a serine protease that catalyzes C3-converting enzyme production. Its levels are significantly reduced in obese and diabetic mouse models. Adipsin enhances glucose-stimulated insulin secretion via the C3a pathway, its knockout in HFD-induced mice results in impaired glucose tolerance (57). FABP4 is associated with metabolism-related CVD mortality and promotes hepatic glucose production (58). Endothelin (endotrophin), secreted by adipose tissue, stimulates fibrosis production and exacerbates HFD-induced local inflammation; its effects are mitigated by endothelin inhibition (59).

#### 2.2.2 Brain

Neural nuclei and networks within the brain orchestrate the integration of key metabolic hormones and neuropeptides from the periphery sources, thereby facilitating adaptive adjustments in food intake and energy expenditure. Central to these processes are the hypothalamic nuclei, which play a pivotal role in energy metabolism. For instance, the arcuate nucleus (ARC) for the hypothalamus, containing both projection and neuroendocrine neurons, releases peptides related to ingestion such as NPY, AgRP, POMC, and dopamine, as well as metabolism-related hormones like growth hormone (60). The regulation of metabolic activities pertinent to NAFLD is primarily mediated through two major pathways: energy intake and energy expenditure.

**Food intake pathways:** Feeding regulation is managed by the hypothalamus through the opposing actions of AgPR and α-melanocyte stimulating hormone (α-MSH). AgRp, released by NPY/AgRP neurons, counteracts α-MSH, produced by POMC neurons (61). α-MSH binds to melanocortin-3 and -4 receptors (MC3R and MC4R), suppressing appetite and promoting catabolic processes. Conversely, in starvation or insufficient energy supply, AgPR inhibits α-MSH, stimulating appetite and increasing food intake (61). In diet-induced obese mice, the absence of G-protein-signaling modulator 1 in POMC neurons leads to improved glucose regulation, enhanced insulin sensitivity, and reduced hepatic steatosis. This is due to increased autophagy and heightened leptin sensitivity via the PI3K/AKT/mechanistic target of rapamycin (mTOR) signaling pathways, which, in turn, boost POMC expression and α-MSH production (62).

Key receptors for the interaction between NPY/AgRP and α-MSH, MC3R, and MC4R are also strongly associated with NAFLD. MC4R deficiency in mice leads to binge eating and obesity (63), and mutation in the MC4R gene in humans is linked to severe early-onset obesity (64). Notably, MC4R-knockout mice fed HFD develop clinical and pathological features resembling human NASH (65). Moreover, hypothalamic MC4R dysfunction has been implicated in IR, dyslipidemia, liver failure, and HCC development. Forcibly shortening MC4R-carrying cilia in hypothalamic neurons impairs neuronal sensitivity to melanocortin, resulting in obesity and leptin resistance (66).

In addition to the ARC, other hypothalamic nuclei contribute to feeding regulation. Damage to the hypothalamic paraventricular nucleus (PVN) in rats leads to overeating and obesity consequences (67). The PVN synthesizes catabolic neuropeptides that promote fatty acid oxidation and lipolysis. Recent studies have shown that forebrain-hypothalamic ER stress-driven circuits mediate hepatic steatosis during obesity, and selective inhibition of PVN ER stress reduces hepatic steatosis in obese individuals (68). Disruption of the ventral medial hypothalamus (VMH) results in binge eating, obesity, and hyperglycemia (69). Neurons in the VMH detect glucose and leptin levels, releasing anorexic neuropeptides such as brain-derived neurotrophic factor (BDNF). BDNF is associated with improved cognitive function and reduced depressive symptoms; reduced levels of BDNF can contribute to binge eating and subsequent obesity (70).

**Energy expenditure pathway:** Energy expenditure is regulated by the sympathetic and parasympathetic nervous system (SNS/PSNS). When the hypothalamic thermoregulatory center senses temperatures below a critical threshold, excitatory neurons in the ventral medial nucleus of the hypothalamus and sympathetic nerves stimulate brown adipose tissue thermogenesis and skeletal muscle activity through norepinephrine and β-adrenergic receptor interactions. The hypothalamic glucose-regulatory center senses low blood glucose levels, subsequently inhibiting insulin secretion and increasing blood glucose via the SNS. Abnormal signals can mislead the hypothalamus into inappropriate energy metabolism adjustments. For instance, co-injected leptin and insulin into the lateral ventricle induces WAT browning and promotes energy expenditure (71). Although the precise role of the PSNS in NAFLD development remains largely undefined, PSNS activation has been shown to significantly reduce hepatic fat accumulation and inflammation in mice subjected to streptozotocin and HFD (72).

The brainstem also plays a critical role in regulating energy homeostasis by receiving local signals from the liver and intestines via extensive vagal innervation. The nucleus of the solitary tract integrates signals from visceral organs and transmits them to higher brain regions (73). In HFD-fed mice and obese individuals, reduced neuronal excitability and disruption of vagal transmission pathways result in impaired hormone activation, such as Cholecystokinin, peptide tyrosine, and glucagon-like peptide-1(GLP-1)) leading to decreased receptor sensitivity (74). This requires stronger stimuli, such as gastric expansion or hormone release, to initiate vagal transmission. Dysbiosis of gut microbiota and resultant endotoxemia are associated with dysfunction in the gut-brain vagal pathway, contributing to obesity and NAFLD.

**Circadian Rhythm Persistence and Periodicity:** Circadian rhythm persistence and periodicity are crucial for maintaining normal blood glucose and lipids levels. The suprachiasmatic nucleus (SCN) is the primary region responsible for generating and regulating circadian rhythm in mammals (75). The SCN controls sugar utilization and protein synthesis through the release of melatonin and glucocorticoids from the central nervous system (76). It generates rhythmic signals that stimulate organs such as the liver to express genes encoding enzymes and transport proteins involved in lipogenesis and lipolysis (77). Disruption in the regular pattern and timing of these circadian rhythms can lead to hyperglycemia and dyslipidemia (78). Furthermore, disruption of the brain and muscle aryl hydrocarbon receptor nuclear translocator-like 1 (BMAL1), a key regulator of biological clocks, has been shown to prevent obesity and metabolic complications associated with HFD. The absence of BMAL1 also inhibits hepatic steatosis and suppresses the expression of differentiation clusters such as CD36 and PPARγ (79).

#### 2.2.3 Gastrointestinal tract

In recent years, the “liver-gut axis”, and “liver-gut microbial axis” have emerged as prominent areas of research in the quest for therapeutic approaches to NAFLD. The gastrointestinal tract, being the principal site for the metabolism and transport of major nutrients, not only provides the precursors necessary for the development of NAFLD but also secretes various signaling molecules that influence intra- and extra-hepatic organ interactions critical to the progression of this disease.

**Growth hormone-releasing peptide (Ghrelin):** Ghrelin, a peptide synthesized in the stomach, exists in two isoforms: acylated ghrelin (AG) and deacylated ghrelin (DAG). In patients with obesity and NAFLD, there is an elevated AG/DAG ratio alongside decreased levels of DAG (80). Increased AG levels are linked to hepatic steatosis in NAFLD patients (81). AG promotes abnormal intrahepatic lipid storage in NAFLD through several mechanisms: it stimulates appetite via the growth hormone secretagogue receptor and activates hypothalamic centers involved in feeding, which boosts caloric intake and induces adipogenesis while decreasing β-oxidation in WAT (82). Additionally, AG through AMPK signaling, encourages TAG storage in the liver, leading to lipid oxidation and mitochondrial dysfunction (83), and modulates interaction between mTOR and PPARγ to further promote adipogenesis (80). Ghrelin also directly facilitates adipogenesis and inhibits lipolysis (83).

Ghrelin may exacerbate NAFLD by advancing the disease to a more severe stage. However, it also acts as an antifibrotic agent by inhibiting hematopoietic stem cell (HSC) activation triggered by fibrotic cytokines such as Transforming growth factor (TGF)-β1 and liver-expressed-antimicrobial peptide 2. Ghrelin’s role in hepatic fibrosis in individuals with obesity and NAFLD is complex, potentially involving a compensatory response to counteract TNF-α induced apoptosis and autophagy (80, 84). Furthermore, Ghrelin has been shown to alleviate TNF-α-induced apoptosis and autophagy in human hepatocytes through AMPK/mTOR pathways (80). Thus, maintaining Ghrelin levels within physiological ranges is crucial, as elevated Ghrelin can impair pancreatic β-cell function and decrease insulin sensitivity (85).

**GLP-1 and glucagon-releasing peptide (GIP):** GLP-1, secreted by intestinal L-cells, enhances insulin release in response to increased glucose levels post-ingestion (86). Besides its role in glycemic control, GLP-1 significantly reduces liver fat accumulation caused by excess lipids and FFAs, thereby hindering NAFLD progression (87). GLP-1 receptor agonists (GLP-1RAs) have been shown to decrease glucose levels, IR, and hepatic lipid content in models of fatty liver. These agents down SREBP-1c and SCD-1 expression in hepatocytes while upregulating PPARα expression, leading to reduced adipogenesis and enhanced free fatty acid β-oxidation (88). Clinical trials have consistently demonstrated that GLP-1RAs effectively reduce hepatic steatosis and improve glycemic control in NAFLD patients. Similarly, GIP produced by proximal small intestinal K cells has been shown to mitigate hepatic steatosis, reduce hepatic inflammation, inhibit hepatic injury, and improve NASH when used in combination with GLP-1RAs (89, 90), though its specific effects on adipose tissue and metabolism remain less clear.

The efficacy of GLP-1 RAs in NAFLD is heterogeneous. While generally improving liver histology and reducing transaminase levels with a favorable safety profile, their effects vary according to specific drug type (91). Liraglutide demonstrates efficacy in reducing hepatic steatosis and hepatocellular ballooning, and may lower gamma-glutamyl transferase (GGT) levels; however, its association with ALT and AST changes is less clear, and its utility in advanced fibrosis remains uncertain, with potential for even promoting fibrosis progression in some cases (91). Conversely, exenatide shows benefit in improving NASH-related fibrosis, correlating with reductions in GGT, ALT, and AST; however, optimal efficacy often requires combination with lifestyle interventions or insulin to address hepatic steatosis (91). Intriguingly, population-based studies comparing GLP-1 RAs with other antihyperglycemic agents reveal a significant association with reduced liver-related mortality but not with NAFLD morbidity, cirrhosis, hepatocellular carcinoma, composite hepatic events, or other hepatic outcomes (92).Further research clarifying the optimal therapeutic range for various GLP-1 RAs subtypes would contribute significantly to optimizing NAFLD management and reducing the associated medication burden.

**Fibroblast Growth Factor (FGF) 19:** FGF19, predominantly expressed in the ileum, regulates systemic lipid and glucose metabolism through endocrine mechanisms (93). By phosphorylating STAT3 and downregulating PPARγ-coactivator 1β expression, FGF19 inhibits lipogenic enzyme expression. It also influences NAFLD development through the bile acid (BA)-farnesoid X receptor (FXR) axis (94).

**Intestinal microbiota:** The human gut harbors the largest and most complex microbial community, playing a pivotal role in inter-organ communication. Research indicates that the composition and density of gut microbiota differ significantly between NAFLD patients and healthy individuals, with a notable correlation between intestinal permeability, bacterial proliferation, and the severity of hepatic steatosis (95).

The gut microbiota’s primary mechanism of inter-organ interaction involves the fermentation of undigested dietary components, leading to the production of metabolites such as short-chain fatty acids (SCFAs). SCFAs are particularly influential in the progression of NAFLD, with elevated levels of total SCFAs commonly observed in NAFLD patients (96). SCFAs affect NAFLD development through multiple pathways: 1) They are absorbed through the intestinal tract, enter the liver via the bloodstream, and contribute to fat synthesis (97). 2) SCFAs stimulate gastrointestinal hormone secretion and, upon binding to G protein-coupled receptors 41 and 43 on intestinal endothelial cells, influence hormone release into the systemic circulation (97); They also bind to free fatty acid receptors 2 and 3, inhibiting Ghrelin activity and enhancing GLP-1 expression (96). By modulating gastrointestinal hormones, SCFAs affect the hypothalamic centers regulating appetite (96). 4) SCFAs promote leptin secretion from adipose tissue, mitigating excessive obesity (97). 5) They inhibit insulin-mediated fat accumulation in the gut, skeletal muscle, adipose tissue, and liver via GPCRs.

Additionally, gut microbes influence NAFLD through BA metabolism regulation. BAs are crucial for cholesterol (CHOL) and lipid breakdown and are involved in metabolic disorders such as obesity, IR, and NAFLD (98). The gut microbiota contributes to BA production and their conversion to various metabolites (99). Modified BAs, particularly hyodeoxycholic acid (HDCA) varieties, negatively correlate with NAFLD presence and severity (100). HDCA enhances probiotic growth and lipolysis through hepatic PPARα signaling, which increases hepatic FXR expression (100). Activated FXR promotes CHOL conversion to BAs, reducing fat accumulation and inflammation, thus alleviating NAFLD (101). HDCA also upregulates hepatic Oxysterol 7-α Hydroxylase (CYP7B1) and inhibits intestinal FXR (100), a unique mechanism that could be pivotal in NAFLD treatment, as CYP7B1 is downregulated in NAFLD, NASH without fibrosis, and T2DM (102).

Recent studies show that CHOL-lowering probiotics improve NAFLD in FXR knockout mice (103) and BA transporter inhibitors (SC-435) increase FGF15 levels, alter BA and microbiota profiles, and improve steatohepatitis in animal models (103). Gut symbionts mitigate metabolic dysfunction-associated steatohepatitis (MASH) through secondary BA biosynthesis (104). These findings underscore the potential of the gut microbiota-liver axis in advancing NAFLD treatment strategies.

The gut microbiota plays a critical role in modulating the innate immune response through its interaction with Toll-like receptors (TLRs). Alterations in gut microbial composition (dysbiosis) and increased intestinal permeability lead to increased translocation of bacterial products, such as lipopolysaccharide (LPS), which act as pathogen-associated molecular patterns (PAMPs). These PAMPs, along with damage-associated molecular patterns (DAMPs) released from injured hepatocytes, engage TLRs, initiating inflammatory signaling cascades. TLRs, pattern recognition receptors, are thus central to the interplay between the gut microbiome and the liver. Specifically, Hepatocyte injury triggers immune cell activation via DAMP-TLR9 signaling (105), while invading pathogens activate TLR pathways through PAMPs (106). Importantly, gut dysbiosis and increased intestinal permeability significantly increase PAMP presentation; conversely, the preservation of the intestinal epithelial barrier by probiotics negatively regulates TLR activation, suppressing the production of pro-inflammatory cytokines such as IL-8 and IL-1β and consequently attenuating NAFLD development (107). This underscores the therapeutic potential of manipulating the gut microbiota to modulate TLR signaling and improve NAFLD.

TLR4 and TLR2 are key receptors mediating interactions with the gut microbiota: TLR2 recognizes Gram-positive bacterial components, while TLR4 recognizes Gram-negative components, such as lipopolysaccharide (LPS) (106). The LPS/TLR4 pathway is a central driver of MAFLD pathogenesis (106). TLR4 activation is not only induced by portal vein LPS accumulation due to gut dysbiosis, but also by hepatic fetuin-A and the obesity-associated adipokine lipocalin-2, further contributing to IR in MAFLD (108, 109). Interestingly, long-chain SFAs, despite lacking direct TLR4 ligand activity, can activate TLR4 signaling and promote inflammation (110). Downstream TLR4 signaling pathways implicated in NAFLD include TLR4/TGF-β1-mediated autophagy (111), LPS/TLR4/FoxO3 signaling in NAFL/NASH (112), and hepatic TLR4-triggered intercellular Jagged1/Notch signaling in NASH-associated fibrosis (113). Antibiotic-mediated reduction of microbial load, or direct TLR4 signaling blockade, can attenuate hepatitis and liver fibrosis (113, 114). Therefore, targeting the LPS/TLR4 pathway presents a promising therapeutic strategy for NAFLD-associated inflammation and fibrosis. Beyond TLR4 and TLR2, other TLRs (TLR3, TLR5, TLR7, TLR8, TLR9), recognizing diverse microbial components (viruses, bacterial proteins, CpG motifs, single-stranded RNAs), may also contribute to NAFLD pathogenesis and inflammatory responses via distinct signaling cascades (106).

#### 2.2.4 Pancreas

The pancreas comprises four primary endocrine cell types (α, β, D, and PP cells) that respond to circulating blood glucose levels and secrete various hormones and peptides influencing NAFLD progression intra- and extra-hepatically. Particularly interesting are the roles of pancreatic β-cells and α-cells in this context.

**Pancreatic β-cells:** Pancreatic β-cells are responsible for insulin secretion, the principal hormone for glucose regulation. IR is a well-established risk factor for NAFLD; however, insulin sensitizers alone are insufficient for NAFLD management (115). Evidence suggests that this inadequacy may stem from impaired β-cell function (116) and a correlation between β-cell dysfunction or compensatory β-cell insufficiency and NAFLD (117, 118). Moreover, β-cells may secrete peptides that modulate NAFLD progression. For instance, islet amyloid from β-cells has been shown to influence glucagon secretion and central satiety signaling and affect blood glucose levels by delaying postprandial gastric emptying (119). Additionally, pancreatic-derived factor secreted from β-cell granules functions as a signaling peptide with a role in glucose and lipid metabolism (120). Thus, β-cell function could serve as a novel diagnostic predictor for NAFLD (118).

**Pancreatic α-cells:** Pancreatic α-cells secrete glucagon, a key regulator of NAFLD development. The “liver-alpha cell axis (LACA)” concept describes how circulating amino acids stimulate α-cells to produce glucagon, which subsequently enhances amino acid metabolism and urea production in the liver (121). Disruption of the hepatic-α-cell axis has been linked to hepatic steatosis and IR (122), and common metabolic disorders such as NAFLD, T2DM, and obesity are known to impair LACA (121). Thus, preserving the hepatic-α cell axis and preventing its disruption by steatosis may offer a novel therapeutic approach for NAFLD (123).

#### 2.2.5 Bone

Beyond its structural and supportive functions, the skeleton acts as an endocrine organ, with osteoblast-derived osteokines significantly influencing MAFLD progression via modulation of hepatic glycolipid metabolism through the bone-liver axis (124). Bone metabolism, critically dependent on the balance between bone resorption and formation, is implicated in MAFLD pathogenesis. This review examines the roles of key bone factors involved in these processes.

**Bone morphogenetic proteins (BMPs):** BMPs regulating various key cytokine signaling pathways (e.g., Wnt, Notch, and FGF) through the TGF-β/BMP signaling cascade, are central to bone and cartilage development and repair (125). Different BMP isoforms exert diverse effects in MAFLD, largely through adipose tissue-liver crosstalk. BMP4, highly expressed in human liver and adipose tissue, correlates with adipocyte size and insulin sensitivity. Conversely, BMP6, upregulated specifically in NAFLD, inhibits HSC activation in murine NASH models, correlating with hepatic steatosis but not inflammation or hepatocyte injury (126, 127). BMP8B deficiency attenuates HSC activation, reduces inflammation, and modifies wound healing, thereby limiting NASH progression; conversely, BMP8B supplementation induces a NASH phenotypes (128). Finally, BMP9, reduced in metabolic syndrome, decreases white adipocyte size and promotes brown adipogenesis, potentially alleviating NAFLD by improving lipid and glucose metabolism via PPARα signaling (129). These findings suggest that BMPs may represent promising therapeutic targets for metabolic syndrome prevention and treatment.

**Osteopontin (OPN):** OPN implicated in biomineralization, bone remodeling, and wound healing, exhibits elevated serum and hepatic levels in NAFLD. In non-obese individuals, elevated serum OPN disrupts hepatic phosphatidylcholine and cholesterol metabolism, contributing to NAFLD progression (130). OPN’s role in NAFLD appears modulated by obesity status, NASH progression, and patient age; OPN upregulation signifies liver injury and fibrosis in NASH, while OPN deficiency mitigates systemic inflammation, inhibits the progression of HCC to less differentiated tumors, and improves overall survival (131). These findings position OPN as a potential therapeutic target in advanced chronic liver disease. Furthermore, OPN acts as an inflammatory cytokine, activating hepatic signaling and promoting M1 macrophage polarization (132). In HFD mice, elevated plasma OPN promotes inflammation and macrophage accumulation in adipose tissue; OPN knockdown reduces adipose tissue inflammation and IR (133), suggesting OPN targeting as a preventative strategy for obesity-associated metabolic diseases like NAFLD and type 2 diabetes (132).

**Vitamin D (VD)**: VD plays a multifaceted role in MAFLD, although its precise effects remain debated. While serum 25-hydroxyvitamin D [25(OH)D] negatively correlates with ghrelin in NAFLD and modulates fatty acid metabolism via the PPARα pathway, ameliorating hepatic steatosis and protecting against advanced fibrosis (134), the active form, 1,25-dihydroxyvitamin D [1,25(OH)_2_D], inhibits MAFLD progression by reducing TAGs, lipid peroxidation, and cellular damage through the SIRT1/AMPK pathway (135). Furthermore, VD_3_ reduces hepatic oxidative stress and inflammation via the SREBP-1c/PPARα-NFκB/IRS2 signaling pathway (136). However, conflicting evidence exists, with some studies showing VD_3_ exacerbation of hepatic steatosis while its analog, calcipotriol, reduces inflammation (137). These discrepancies may stem from variations in VD levels, forms, and selective VDR activation, along with the restoration of mitochondrial contact sites (137, 138). Despite this complexity, randomized controlled trials demonstrate the beneficial effects of high-dose cholecalciferol on serum ALT, hsCRP, and lipid profiles in NAFLD patients (139, 140), highlighting the need for further investigation into the diverse regulatory mechanisms of different VD forms and levels in MAFLD pathogenesis.

**Other bone factors:** summarizing the relevant hepatic bone crosstalk and NAFLD related reviews in the past two years, and summarizing the bone factors in them (**Table 1**).

**Table 1:** Mechanism of action of bone factor in NAFLD.

Bone factor	Organs	Main mechanism	Diseases involved	Reference
FGF 23	Bone kidney Hepatic	Interferes with osteoblast differentiation and matrix mineralization, Promotes renal phosphate excretion, Promotes liver inflammation	hypophosphatemia-related rickets; ADHR CKD MAFLD	(124)
Osteocalcin	Bone Hepatic	Determinants of bone formation, Increases insulin secretion and sensitivity, Inhibits NAFLD phenotype	Metabolic Bone Disease MASH	(124)
Calcitonin	Bone Adipose tissue Hepatic	Inhibits bone resorption, promotes lipid accumulation, promotes liver fibrosis	Paget’s disease Osteoporosis Obesity MAFLD	(124)
Sclerostin	Bone Hepatic Adipose tissue	Inhibits bone formation, promotes lipid accumulation, promotes insulin resistance	Osteoporosis MAFLD	(124)
Lipocalin 2	Bone Hepatic Brain	Promotes osteogenic differentiation, liver inflammation, appetite suppression	MAFLD Obesity Alcoholic Hepatitis	(124)
TGF-β	Bone Hepatic	Promotes bone resorption, promotes liver fibrosis, inhibits hepatocellular carcinoma in early stage, promotes hepatocellular carcinoma in late-stage	MAFLD HCC	(124)
Insulin-like growth factor-1	Bone Hepatic	Regulation of bone homeostasis and longitudinal bone growth during pre-puberty and puberty and maintenance of bone mass during adulthood	Osteoporosis MAFLD	(178)
FGF21	Bone Pancreatic WAT Hepatic	Promotes bone resorption, enhances pancreatic β-oxidation, ketogenesis, and gluconeogenesis, promotes WAT browning, independently predicts hepatic steatosis	Osteoporosis MAFLD/MASH T2MD	(178)
N-terminal propeptide of procollagen type 1 (P1NP)	Bone Hepatic	Precursor of type 1 collagen, closely related to liver fibrosis	Metabolic Bone Disease Cirrhosis of the liver in the compensated phase	(179)
Osteoprotegerin (OPG)	Bone Hepatic cardiovascular	Inhibition of osteoclast activation and stimulation of apoptosis to promote fibrosis	metabolic syndrome MAFLD Progressive Atherosclerotic Cardiovascular Disease	(179)

**Osteoporosis (OP)**: The co-occurrence of OP and NAFLD is well-established, yet the precise nature and mechanisms underlying their interaction remain unclear. While recent studies (within the past two years) suggest a causal link between NAFLD and OP (141, 142), the reverse causality remains less defined.

The observed correlation between NAFLD and OP is likely influenced by several factors, including age, sex, ethnicity, bone site-specific bone mineral density (BMD), and the severity and co-morbidities associated with NAFLD (143). Cross-sectional studies reveal a complex picture: BMD in younger patients appears unaffected by steatosis or non-alcoholic steatohepatitis (NASH) (144), while in children, reduced BMD correlates with the histological severity of NAFLD, particularly NASH (144). In contrast, adult studies reveal a negative correlation between BMD and advanced hepatic fibrosis in diabetic men aged ≥50 years with MAFLD, with increased spinal fracture risk associated with advanced fibrosis in men ≥40 years (145, 146);. Furthermore, in women, menopausal age significantly influences OP risk in the context of NAFLD, demonstrating a negative correlation between BMD and advanced hepatic fibrosis in postmenopausal diabetic women with MAFLD (145, 147). These findings highlight the need for further investigation to elucidate the complex interplay between NAFLD and OP across different demographic groups and disease stages.

The relationship between MAFLD-associated hepatic fibrosis and the risk of reduced BMD and osteoporosis in T2DM patients may be significantly influenced by obesity. Studies suggest that the observed correlation disappears when analyzed independently of obesity, potentially due to the strong association between NAFLD-related hepatic fibrosis, lower BMD, and impaired bone microarchitecture in obese individuals (148, 149). Furthermore, ethnic disparities exist, with differences in BMD and osteoporotic fracture risk observed between Asian and non-Asian populations, possibly reflecting underlying skeletal differences (150). Epidemiological data, such as the 2017-2018 US National Health and Nutrition Examination Survey (NHANES) analysis, show a negative correlation between NAFLD and lumbar spine BMD (151), while other contemporary analyses have demonstrated a causal link between NAFLD and forearm BMD (152). Despite the ongoing debate regarding the precise nature of the NAFLD-OP association, the available evidence underscores the need for individualized preventative and therapeutic strategies tailored to specific patient populations presenting with co-morbid NAFLD and OP.

#### 2.2.6 Skeletal muscle

Skeletal muscle, a crucial organ for energy expenditure and thermogenesis, is intricately linked to metabolic diseases. Petermann-Rocha et al. found in a cross-sectional study that reduced muscle mass and strength exacerbate NAFLD over time (153). Similarly, Iwaki et al. demonstrated that sarcopenia not only increases the risk of NAFLD but also accelerates its progression (154). This phenomenon is further elucidated by studies indicating that elevated levels of muscle growth inhibitory hormones—myokines responsible for muscle atrophy—are associated with poorer survival rates in cirrhotic patients (155). Consequently, considerable research has focused on identifying specific myokines and their signaling pathways as potential therapeutic targets for NAFLD.

Exercise and dietary modifications are established strategies for preventing and delaying NAFLD. Given its unique role in locomotion, skeletal muscle has garnered significant attention. During physical activity, skeletal muscle secretes various myokines and generates numerous metabolites, though only a subset of these have been implicated in inter-organ communication relevant to NAFLD (156).

Exercise-induced activation of PGC-1α prompts skeletal muscle to release irisin. Irisin not only induces the browning of WAT and enhances energy metabolism (157), but also interacts with Myeloid differentiation factor 2 (MD2), thereby blocking the MD2-TLR4 interaction (158). This action inhibits downstream MAPK and NF-κB pathways, preventing the release of pro-inflammatory factors. Furthermore, exercise-induced PGC-1α regulates the release of muscle-derived amino acid metabolites such as β-aminoisobutyric acid (159) and 3-hydroxybutyric acid, which influence NAFLD by modulating lipid synthesis and fatty acid oxidation processes (160). Additionally, FGF21 produced in response to exercise promotes lipolysis of LDs, mitigates LD accumulation, and attenuates NAFLD while slowing hepatic senescence through the AMPK/UNC-51-like kinase 1 (ULK1) pathway (161). FGF21 also enhances glucose utilization in skeletal muscle, contributing to increased energy expenditure (162).

Exercise-induced metabolites impact NAFLD through their effects on both skeletal muscle and adipose tissue. For example, IL-15 fosters muscle hypertrophy, while leukemia inhibitory factor (LIF) supports muscle regeneration and influences NAFLD development through muscle remodeling (163). Similarly, cysteine-rich acid secretory protein inhibits adipogenesis, IL-6 promotes lipolysis, and BDNF activates the AMPK pathway in adipose tissue, thus controlling lipid oxidation and reducing NAFLD (163).

The role of skeletal muscle in NAFLD progression underscores the potential of targeting skeletal muscle-derived myokines to influence disease outcomes. For instance, skeletal muscle-specific IRF4 knockout mice fed a NASH diet showed improvements in hepatic steatosis, inflammation, and fibrosis, despite no change in body weight. This effect is mediated by IRF4’s regulation of the myokine follicle-stimulating hormone-like protein 1 (164).

Despite the promise of myokines as therapeutic agents for NAFLD, challenges remain. Many myokines share similarities with adipose-derived factors, raising the risk of misclassification when determining their functions (165). Moreover, the variation in myokine levels in human myofibroblasts or NAFLD populations is minimal, complicating efforts to directly demonstrate their effects on NAFLD.

While the application of exercise interventions in NAFLD management presents challenges, various exercise modalities offer distinct therapeutic benefits. Aerobic training (AT) effectively reduces hepatic steatosis and systemic inflammation, potentially improving histological markers in MAFLD (166, 167). Resistance training (RT) enhances insulin sensitivity and promotes muscle hypertrophy, leading to improvements in cardiac and hepatic function (168, 169). High-intensity interval training (HIIT) offers unique metabolic advantages, including enhanced mitochondrial function, improved whole-body lipid metabolism, increased exercise capacity, and enhanced peripheral insulin sensitivity, demonstrating safety and efficacy in NASH (170, 171). A comparative analysis of these modalities is crucial for optimizing NAFLD treatment strategies.

Comparative analyses reveal modality-specific benefits. AT demonstrates superior efficacy in lowering ALT and AST levels, making it particularly suitable for overweight individuals, older adults, pregnant women, and those with arthritis due to its low-impact nature and enhancement of cardiorespiratory fitness (172). RT provides a viable alternative for individuals unable to tolerate AT, effectively improving AST levels and promoting muscle strength. HIIT, while also effective in reducing ALT and AST, appeals to younger, healthier individuals, athletes, and some patients with chronic conditions owing to its efficiency and flexibility (173). While HIIT improves cardiorespiratory fitness and metabolism, integrated programs combining AT and RT show superior efficacy in improving lipid profiles, specifically lowering TC and TG, while raising HDL-C and lowering LDL-C (174). This integrated approach may represent the optimal strategy for maximizing physical conditioning, improving cardiovascular health, and reducing hepatic fat accumulation in NAFLD patients (174). Ultimately, a personalized approach to exercise prescription, tailored to individual needs and capabilities, is essential for optimizing both health outcomes and quality of life.

## 3 Conclusion and future directions

**Figure 2:**
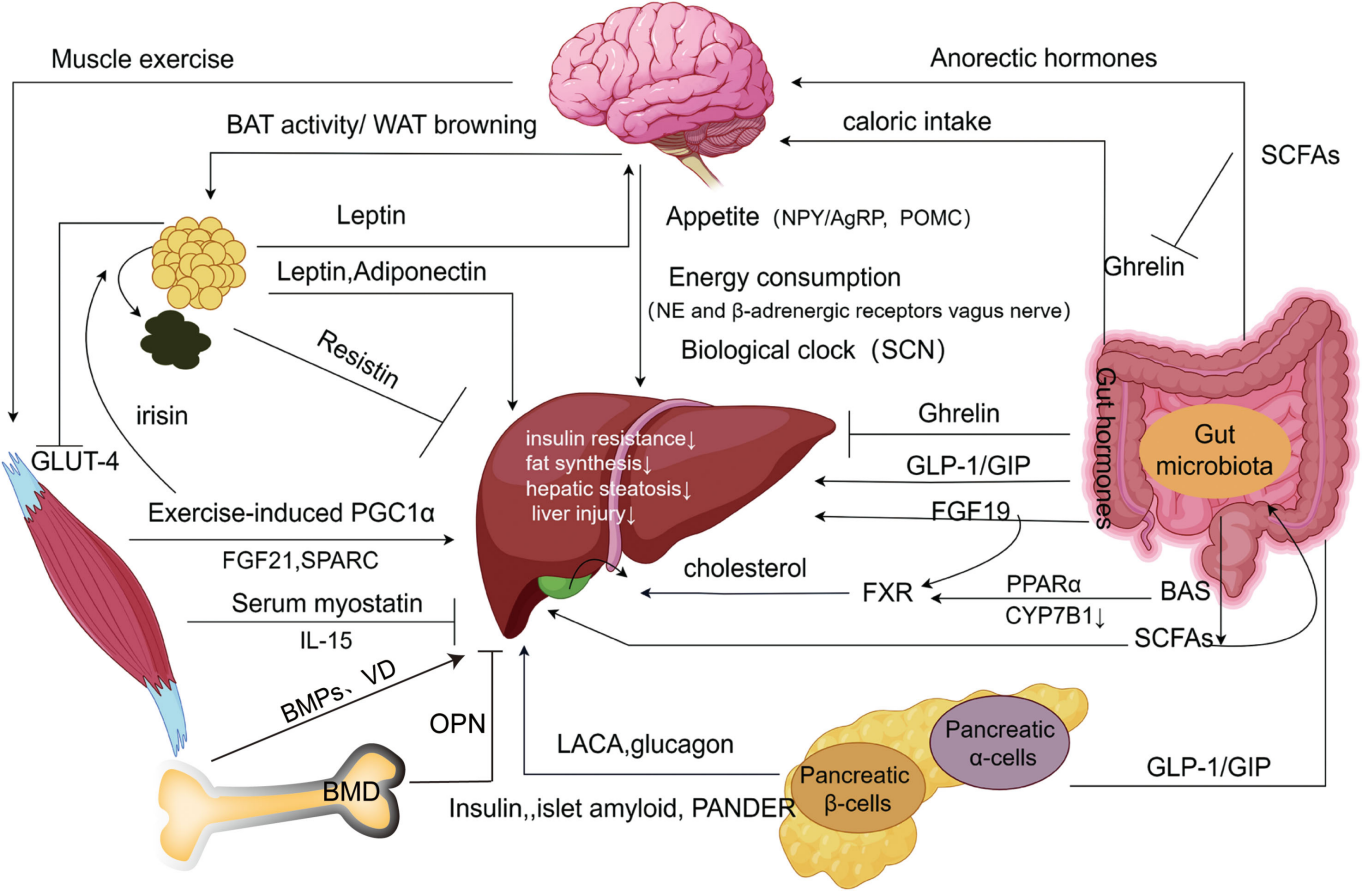
Extrahepatic organ-specific networks associated with NAFLD. Adipose tissue produces adipokines that inhibit hepatic steatosis, inhibit hepatic inflammatory injury, and increase insulin sensitivity through “the hepatic-fatty axis”, “hepatic-fatty-cerebral axis”, and “hepatic-fatty-skeletal muscle axis”, which may be a form of feedback regulation. The brain receives metabolism-related signals from external organs (adipose tissue, gastrointestinal tract, etc.), to improve impaired NAFLD via food intake, energy expenditure, and biological clock pathways. The gastrointestinal tract influences extrahepatic organs such as the brain and pancreas to inhibit the development of NAFLD by releasing intestinal hormones with associated peptide metabolism, while gut microbes have a similar effect by altering short-chain fatty acids and bile acids. The endocrine pancreas improves insulin resistance and inhibits steatosis through the “hepatic-alpha-cell axis”, and “hepatic-β-cells axis”. Exercise-induced skeletal muscle production of myokines and metabolites promotes white fat burning in extrahepatic adipose tissue and inhibits intrahepatic lipogenesis, inhibiting the progression of NAFLD. Bone metabolism produces bone factors that affect hepatic steatosis, hepatitis, and liver fibrosis through the “liver-bone axis”, and with the change in bone density, the development of NAFLD will also be affected. SCN, Suprachiasmatic nucleus; NPY/AgRP, Neuropeptide Y/Agouti-related protein; PPARα, Peroxisome proliferator-activated receptor α; GLP-1, Glucagon-like peptide-1; GIP, Glucagon-releasing peptide; SCFAs, Short-chain fatty acids; CYP7B1, Hepatic Oxysterol 7-α Hydroxylase; LACA, Liver-alpha cell axis; WAT, White adipose tissue; BAT, Brown adipose tissue; SPARC, Cysteine-rich acid secretory protein. BMPs, Bone morphogenetic proteins; OPN, Osteobridging proteins; VD, Vitamin D; BMD, Bone mineral density (Drawing by Figdraw 2.0).

Undoubtedly, NAFLD (or MAFLD) has a complicated etiology that includes coordinated regulation of several organs. These organs exert direct influences (e.g., leptin inhibiting hepatic fat storage), indirect effects (e.g., leptin influencing appetite and reducing food intake), and the activation of the same molecules across different signaling pathways, collectively forming intricate organ networks (**Figure 2**). These insights provide additional targets for understanding and treating NAFLD.

This review analyzed clinical trials (Phase I-III) related to NAFLD (**Table 2**), revealing a predominant focus on intrahepatic signaling pathways and the hepato-intestinal axis in current drug development efforts. While diet and exercise remain cornerstones of NAFLD management, the efficacy of combined interventions, particularly GLP-1 RAs in conjunction with other therapies, has shown promise (**Table 3**). Future research should prioritize combination therapies targeting both the liver-gut axis and the emerging liver-gut-bone/skeletal muscle axis, while continuing to explore novel therapeutic targets. However, the potential therapeutic value of other extrahepatic metabolic factors warrants further investigation (**Table 4**).

**Table 2:** NAFLD-related clinical trials in the last 3 years.

Interventions	Clinical phase	Target	Consequences	Reference
ZSP1601	Ib/IIa NCT04140123	Pan phosphodiesterase inhibitor	Effective and well-tolerated improvement in liver chemistry, liver fat content, and fibrosis in NAFLD patients	(180)
BFKB8488A	Ib NCT03060538	Fibroblast growth factor receptor 1/Klothoβ agonist	Lower triglycerides, improved HDL, reduced liver fat in NAFLD patients, well tolerated in patients with T2DM or NAFLD	(181)
pegozafermin (BIO89-100)	Ib/IIa NCT04048135 NCT04929483 NCT0441186	FGF21 analog	Reduces liver fat and improves liver fibrosis. Usually well tolerated	(182–184)
Survodutide	I NCT05296733	Glucagon/GLP-1 receptor dual agonist	Often tolerated in patients with compensated or decompensated cirrhosis	(185)
FGF401 ,/+spartalizumab	I/II NCT02325739	FGF19-FGFR4 signaling	Increased levels of total bile acids and circulating FGF19 are safe in patients with FGFR4/KLB-positive tumors	(186)
BI 685509	Ib NCT03842761	Soluble guanylate cyclase activator	Usually well tolerated	(187)
TQA3526	I ChiCTR1800019570	FXR agonist	Increases circulating FGF-19 and decreases bile acid precursor C4 levels, well tolerated	(188)
EDP-297	I NCT04559126	FXR agonist	Presence of severe cases, discontinuation of medication	(189)
Efruxifermin	IIb NCT04767529	FGF21 analog	Improvement in hepatic fibrosis with serious adverse events	(190)
vonafexor	IIa NCT03812029	FXR agonist	Improve liver enzymes, Lose Weight, and Gain Kidney Benefits safely and effectively	(191)
Aldafermin	II b NCT03912532	FGF19 analog	Overall well tolerated, may alter liver fibrosis	(192)
Efinopegdutide	IIa NCT04944992	Glucagon/GLP-1 receptor dual agonist	Reducing liver fat content in NAFLD patients is safe and effective in treating NASH	(193)
Tropifexor	II NCT02855164	FXR agonist	Lower liver fat, safe and effective for NASH	(194)
Licogliflozin	II a NCT03205150	Inhibition of sodium-glucose cotransporter proteins 1 and 2	Reduced serum alanine aminotransferase in NASH patients without serious adverse events	(195)
Denifanstat	II b NCT04906421	FASN inhibitor	Inhibits MASH inflammation and fibrosis without serious adverse events	(196)
Ervogastat /+clesacostat	II NCT04321031	DGAT2i+ACCi	Reduce liver fat, inhibit NASH inflammation	(197)
PF-06835919	IIa	Ketose kinase inhibitor	Reduces liver fat in patients with NAFLD and T2D safely and effectively.	(198)
Cenicriviroc	III NCT03028740	Chemokine receptor type 2 and type 5 antagonists	safe and effective, but no antifibrotic efficacy has been seen in adult NASH patients	(199)
Obeticholic Acid	III NCT02548351	Bile Acid Receptors	Improvement of hepatic fibrosis, suppression of hepatic inflammation, presence of discontinuation of treatment due to itching	(200)
Semaglutide	II III NCT03987451 NCT02970942 NCT04822181	GLP-1Ras	Improvement of hepatic steatosis and dose-related effects on liver fibrosis	(201–203)
Resmetirom	III NCT04197479 NCT03900429	Thyroid hormone receptor β	Suppresses liver fat, liver inflammation, improves liver fibrosis and is safe and effective	(5, 6)

Underlined text is the Trial registration number.

**Table 3:** Clinical trials in the last 3 years on combined interventions for NAFLD.

Joint interventions	Type of intervention	Event	Reference
Progressive RT (PRT) + Weight Loss	Exercise + Weight Loss	PRT Did Not Enhance Weight Loss Benefits for NAFLD	ACTRN12622000640707 (204)
FGF401 +spartalizumab	FGF19+PD-1	Increased levels of total bile acids and circulating FGF19 are safe in patients with FGFR4/KLB-positive tumors	NCT02325739 (186)
Semaglutide +cilofexor + firsocostat	GLP-1RAAs+FXR+ ACCi	More effective in the treatment of hepatic steatosis and biochemical indicators than semaglutide alone	NCT03987074 (205)
Ervogastat/ +clesacostat	DGAT2i+ACCi	Reduce liver fat, inhibit NASH inflammation	NCT04321031 (197)
Efruxifermin + GLR-1Ras	GLP-1RAs+FGF21	Reduced hepatic fibrosis in MASH and T2D patients compared to GLR-1Ras with comparable tolerability	NCT05039450 (206)
Saroglitazar + Vitamin E	PPAR-α/γ agonists + antioxidants	Combination therapy improves insulin resistance and increases HDL levels compared to treatment alone	CTRI/2022/01/039538 (207)
Every other day fasting +AT	Diet + Exercise	Combination Group Reduces Body Weight and Increases Insulin Sensitivity, but Does Not Enhance Benefits for Hepatic Steatosis in NAFLD Patients	(208, 209)
Dietary Changes + Aerobic Exercise	Diet + Exercise + Gut Microbes	Host-Gut Microbiome Ecosystems Provide a Pathway for Developing Personalized Intervention Strategies for Treating NAFLD	(210)
Semaglutide + Empagliflozin	GLP-1RAs+ SGLT-2	Completion in 2025	ChiCTR2300070674 (211)
Tofogliflozin + Pioglitazone	SGLT-2+ PPAR-γ agonist	Both cumulative effects of single drugs and cardioprotective effects	(212)
Mediterranean diet + intermittent fasting	Dietary changes	Existence of Time Limits May Improve Steatosis in NAFLD Patients in the Long Term	(213)

Underlined text is the Trial registration number.

**Table 4:** Summary of the role of metabolic factors in NAFLD.

Main metabolicfactors	Relevant organ(s)	Main mechanisms	Applications
Leptin	Adipose tissue Brain Hepatic	Suppresses appetite and promotes glycolipid metabolism	Evaluating the NAFLD condition
Adiponectin	Adipose tissue muscle Hepatic	Increase insulin sensitivity, promote lipid metabolism, anti-atherosclerosis	Screening for people at high risk for NAFLD
Resistin	Adipose tissue muscle Hepatic	Promotes inflammation and insulin resistance	Providing a reference point for the diagnosis of NAFLD
α-MSH	Brain Hepatic	Suppresses appetite and stimulates metabolism	Modifying Diet to Control NAFLD
AgRp	Brain Hepatic	Stimulates appetite and increases food intake	Modifying Diet to Control NAFLD
BDNF	Brain Hepatic	Regulation of food intake	Control of obesity due to overeating and binge eating
BMAL1	Brain Hepatic	Regulating the persistence and periodicity of circadian rhythms	Prevention of obesity and metabolic complications associated with a high-fat diet
Ghrelin	Brain Gastrointestinal tract Pancreatic Hepatic	Promotes appetite and lipogenesis	Maintain normal levels to prevent damage to pancreatic function
GLP-1	Gastrointestinal tract Hepatic	Regulates blood glucose and improves liver histology	Good clinical feedback exists for the treatment of NAFLD with GLP-1Ras drugs
FGF19	Gastrointestinal tract Hepatic	Regulates systemic lipid and glucose metabolism	Specific targets for drug discovery
FXR	Gastrointestinal tract Hepatic	Regulates bile acid metabolism and inhibits liver inflammation	Specific targets for drug discovery
BAs	Gastrointestinal tract Hepatic	Modifying gut microbes to regulate cholesterol metabolism	Specific targets for drug discovery
TLRs	Gastrointestinal tract Hepatic	Participates in innate immunity and promotes liver inflammation	Good feedback exists for gut microbial therapy
BMPs	Bone Adipose tissue Hepatic	Improve glycolipid metabolism and inhibit inflammation	Prevention of metabolic syndrome
OPN	Bone Hepatic	Promotes liver injury and fibrosis	Therapeutic targets for advanced chronic liver disease
VD	Bone Hepatic	Improvement of hepatic steatosis and prevention of advanced fibrosis	Adjuvant NAFLD treatment
Irisin	Skeletal muscle Hepatic	Regulates lipid synthesis and fatty acid oxidation	Cue the beneficial effects of exercise in NAFLD
FGF21	Skeletal muscle Hepatic	Promotes energy expenditure and slows down liver aging	Specific targets for drug discovery

Furthermore, new research shows that over 55% of people with T2DM also have NAFLD (175), which is a definitive pathogenic factor for CKD, significantly increasing CKD risk among MAFLD patients (176). NAFLD patients face elevated risks of cardiovascular events, with ASCVD being a primary cause of mortality (177). Exploring mechanisms of inter-organ crosstalk not only offers new perspectives for developing NAFLD therapies but also identifies novel targets for intervening in other metabolic-related extra-liver diseases.
